# Estimation of pack density in grey wolf (*Canis lupus*) by applying spatially explicit capture-recapture models to camera trap data supported by genetic monitoring

**DOI:** 10.1186/s12983-018-0281-x

**Published:** 2018-10-03

**Authors:** Luca Mattioli, Antonio Canu, Daniela Passilongo, Massimo Scandura, Marco Apollonio

**Affiliations:** 1 Settore Attività Faunistico Venatoria, Pesca Dilettantistica, Pesca in mare, Regione Toscana, Via A. Testa 2, I–52100 Arezzo, Italy; 2 Department of Veterinary Medicine, University of Sassari, via Vienna 2, I-07100 Sassari, Italy

**Keywords:** Camera trapping, *Canis lupus*, Individual recognition, Non-invasive genetic sampling, Pack density estimation, Pack size, SCR, Spatially explicit capture-recapture models

## Abstract

**Background:**

Density estimation is a key issue in wildlife management but is particularly challenging and labour-intensive for elusive species. Recently developed approaches based on remotely collected data and capture-recapture models, though representing a valid alternative to more traditional methods, have found little application to species with limited morphological variation. We implemented a camera trap capture-recapture study to survey wolf packs in a 560-km^2^ area of Central Italy. Individual recognition of focal animals (alpha) in the packs was possible by relying on morphological and behavioural traits and was validated by non-invasive genotyping and inter-observer agreement tests. Two types (Bayesian and likelihood-based) of spatially explicit capture-recapture (SCR) models were fitted on wolf pack capture histories, thus obtaining an estimation of pack density in the area.

**Results:**

In two sessions of camera trapping surveys (2014 and 2015), we detected a maximum of 12 wolf packs. A Bayesian model implementing a half-normal detection function without a trap-specific response provided the most robust result, corresponding to a density of 1.21 ± 0.27 packs/100 km^2^ in 2015. Average pack size varied from 3.40 (summer 2014, excluding pups and lone-transient wolves) to 4.17 (late winter-spring 2015, excluding lone-transient wolves).

**Conclusions:**

We applied for the first time a camera-based SCR approach in wolves, providing the first robust estimate of wolf pack density for an area of Italy. We showed that this method is applicable to wolves under the following conditions: *i*) the existence of sufficient phenotypic/behavioural variation and the recognition of focal individuals (i.e. alpha, verified by non-invasive genotyping); *ii*) the investigated area is sufficiently large to include a minimum number of packs (ideally 10); *iii*) a pilot study is carried out to pursue an adequate sampling design and to train operators on individual wolf recognition. We believe that replicating this approach in other areas can allow for an assessment of density variation across the wolf range and would provide a reliable reference parameter for ecological studies.

**Electronic supplementary material:**

The online version of this article (10.1186/s12983-018-0281-x) contains supplementary material, which is available to authorized users.

## Background

Since the 90s a growing scientific literature has addressed the role of apex predators in structuring animal communities, maintaining biodiversity and ecosystems services through complex trophic cascades (for a review see [1, 2]). Consequently, obtaining accurate estimates of abundance and density of such predators has become a key issue in ecosystems management. Moreover, density of large predators may serve as a meaningful bioindicator, being related to the abundance of large prey, which is often difficult to estimate. Large predators are difficult to survey, due to their low density, their elusive behaviour and frequent preference for closed habitats [3]. Many traditional field techniques like snow or ground tracking, sound recording or searching for other field signs (i.e. scats, remainders of predation) require a big effort and entail difficulties in data interpretation because of the lack of individual recognition. Radio tracking is a more effective technique, but requires huge budgets to obtain a sufficient sample size and is more invasive for animals, as it implies capture and manipulation. In the past decade, advances in technology and data modelling offered new tools to biologists to pursue the important goal of estimating predators’ population size. In particular, non-invasive genetic sampling (NGS) and camera trapping (CT), combined with capture-recapture (CR) models, revolutionized field studies on many large terrestrial predators in forested habitats [4], as they allow individual recognition and the obtainment of encounter history data without animal manipulation. NGS has been largely applied in monitoring programs of carnivore populations, since it can provide a set of outstanding information such as species, gender and individual determination, kinship, dispersal and hybridization (for a review see [5, 6]). Additionally, NGS can also provide data for capture-recapture estimation of population abundance, though its use is constrained by the field effort and laboratory costs that are necessary to collect and analyse an adequate number of fresh samples (usually scats or hairs). For these reasons, population abundance estimation by NGS is more frequently used in large-scale monitoring projects with large budgets, or when dealing with species that are not easily recognizable by photo-identification [7, 8]. CT was mainly used in monitoring felid species whose coat patterns allow for an unambiguous individual identification, e.g. tiger (*Panthera tigris* [9]), leopard (*Panthera pardus* [10, 11]), snow leopard (*Panthera uncia* [12]), jaguar (*Panthera onca* [13]), Eurasian lynx (*Lynx lynx* [14]) and ocelot (*Leopardus pardalis* [15]). Nevertheless, in the last few years, CT has been increasingly used also for species lacking evident natural markings, like puma (*Puma concolor* [16]), lowland tapir (*Tapirus terrestris* [17]), forest elephant (*Loxodonta cyclotis* [18]) chimpanzee (*Pan troglodytes* [19]), and also some canids (coyote *Canis latrans* [20]; maned wolf *Chrysocyon brachyurus* [21] and red fox *Vulpes vulpes* [22]). In these studies, individual recognition was obtained from different morphological traits like tail shape and carriage or fur colour markings in specific areas (e.g. head, legs or tail).

Despite the wolf is one of the most studied large predators worldwide, no study has so far reported abundance and/or density estimation of wolves by camera traps. In a pilot investigation, Galaverni and colleagues [23] combined CT with NGS to test their effectiveness in monitoring a wolf population. The authors stated that, although identifying individual wolves from photographic material during the study was rarely possible, CT data allowed an estimation of the minimum packs size that was similar to that obtained through NGS. Actually, the main issue related to the use of a camera-trap capture-recapture (CTCR) approach with wolves primarily concerns the presence of sufficient phenotypic variation for individual recognition. Although wolves lack evident natural markings, external idiosyncrasies (e.g. permanent injuries, blind eyes) often occur in a population, allowing for individual recognition. Moreover, wolf-dog hybridization events, documented in many areas of Europe [24], can introduce phenotypic variation in traits like body size, pelage colour, length, shape and carriage of tail and ears. This source of morphological variation, possibly combined with individual difference in behavioural traits associated to social status (e.g. scent-marking display), can allow individual recognition in wolf populations.

A second issue is the applicability of CR approaches to group-living species, given that CR models assume uncorrelated activity centres of individuals (i.e., independence of capture events [4]. As wolves live in packs, capture events are often correlated, violating this assumption. For these reasons, the adoption of a CTCR method in wolves is challenging and needs further validation. Moreover, accurate estimates of wolf density are very infrequent in Europe and limited to a few radio-tracked wolf populations [25–27]. In Italy, estimates of wolf population density are scarce [28, 29], whereas a large amount of grey literature reporting on local abundance was used to extrapolate large-scale density values [30, 31]. In this study, we tested for the first time the applicability of CT to obtain robust estimates of wolf density using CR methods. We chose a pack-based approach, inferring total wolf density from pack density and average pack size as a conversion factor [32]. We studied an Italian wolf population, where individual recognition was facilitated by the introgression of canine genes [33] and validated by the support of NGS data coming from a long-term research project. Our aims were (i) to obtain a robust density estimate for the wolf population of our research area in the province of Arezzo, Central Italy, comparing a Bayesian approach with a likelihood based one; (ii) to test for repeatability of our method by evaluating the effect of inter-observer disagreement in wolf identification on density estimates; and (iii) to test the effect of different survey periods and CT sampling design on wolf density estimate. Finally, we discuss our results in the light of the up-to-date knowledge on wolf density at local and continental scale, and evaluate pros and cons of the application of a CR approach on camera trapping data in this species.

## Results

### Wolf recognition

**Fig. 1:**
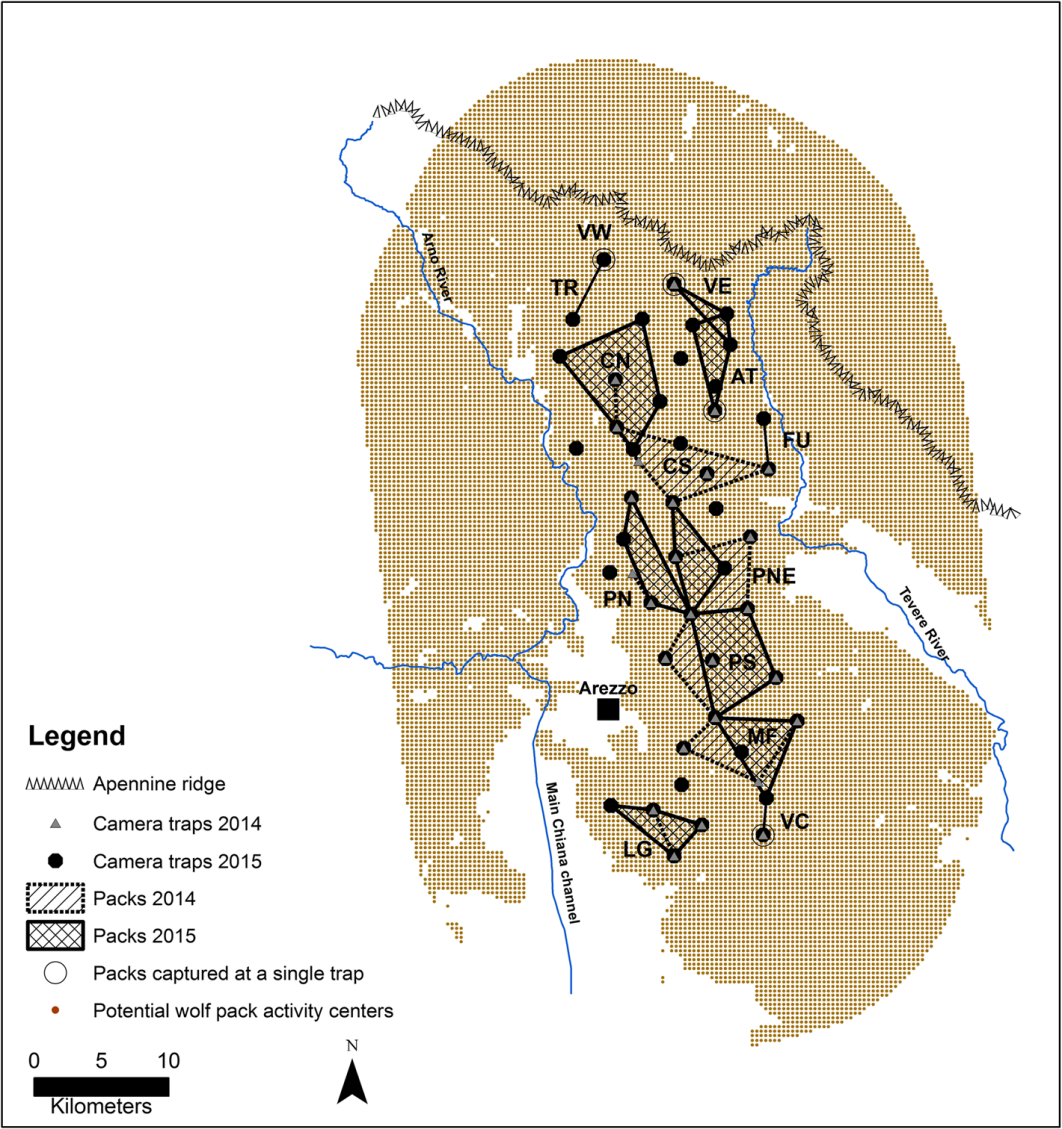
Camera trapping study area in Arezzo province (Italy), where wolf density was estimated in 2014 and 2015. Approximate locations of the 13 detected packs were reconstructed by the video-captures of focal animals at distinct trap sites during the study sessions. The large dotted area is the habitat suitable to wolves and is formed by dots representing potential wolf pack activity centres (spaced 666 m and buffering the trap array by a 15-km radius). Unsuitable habitats for wolf were excluded from calculations and are shown in white

During 303 sampling occasions (5197 trap days), from the 1st of April 2014 to the 11th of June 2015, we achieved a total of 909 wolf videos corresponding to 657 independent capture events (CE) (1.38 videos/CE) and 1240 individuals captured (1.89 individuals/CE). In 130 CE, wolf recognition was not possible because of low video quality (e.g. fuzzy videos of running individuals, recording only part of the silhouette, excessive distance of the individual from the camera, or bad weather conditions). Of the remaining 527 usable CE, 427 (81.0%) were assigned to a specific pack and the remaining 100 were classified as undetermined. One or both members of the breeding pair were captured in 356 out of these 427 CE (54.2% of the total) and, after having selected one single focal individual/pack, its relative capture histories were created (in total 295 CE). The data of the pilot study and the two sessions are summarized in Table 1. During the pilot study and the first session (2014), we identified 10 packs constituted of 20 alpha (α), 14 beta (β) and 9 pups for a total of 43 wolves (see Additional file 1). In the subsequent session (2015), 9 out of 10 packs were confirmed and 3 new packs were identified, two of which at trap sites that were not used in 2014 (Fig. 1), for a total of 12 different packs. In the 2015 session we ascertained the presence of 50 wolves, 24 of which were α and 26 were β (the latter including individuals born in 2014). In total, 14 different focal individuals were considered: 10 individuals belonged to the packs observed in the 2014 survey, three individuals to three new packs discovered in 2015 and the last one was the new α male M22 that in 2015 replaced the α male detected in one of the 2014 packs (Table 2). The distinctive features used for their recognition are described in Additional file 1. A selection of the video captures of each focal animal used for density calculations is available in Additional file 2.

**Table 1:** Camera trapping sampling design and outcome of the pilot study and the two sessions (2014 and 2015) carried out in the study area (Arezzo province, Italy)

	Pilot Study	Session 2014	Session 2015	Total
Time period	1 April - 18 June 2014	19 June – 28 August 2014	10 January – 11 June 2015	
MCP trap array (Km^2^)	–	414	560	
Camera stations	7–20	26	45	
Sampling occasions (days)	79	71	153	303
Mean sampling occasion/trap (SD)		58.9 (12.5)	55.9 (12.6)	
Trap days	1094	1533	2570	5197
Wolf videos	194	265	450	909
Independent capture events (CE)	147	188	322	657
CE/100 trap days	13.44	12.26	12.53	12.64
Unusable CE^a^	31	24	75	130
Unassigned CE^b^	26	21	53	100
CE assigned to a pack	90	143	194	427
CE with focal animal (used)	69	98	128	295

^a^Low quality videos (wolves far from the camera, passing without stopping, partially captured, presence of fog)

^b^Good quality videos (but no focal animal identified)

**Table 2:** Summary of capture history data collected for 14 focal animals belonging to 13 different packs during camera trapping sessions 2014 and 2015 (Arezzo province, Italy)

		Session 2014		Session 2015		
Focal animal	Pack	Independent captures	Distinct traps	Independent captures	Distinct traps	Last video capture
F1	PS	13	6	13	5	04/08/15
F6	PNE	12	5	10	5	02/09/16
F12	LG	8	3	11	4	25/05/16
F20	VE	15	1	14	3	27/08/16
F21	AT	1	1	18	5	20/09/16
M6	PN	12	2	14	4	30/08/16
M8	VC	5	1	3	2	20/02/15
M15	CN	3	2	17	6	25/09/16
M14	CS	8	4	–	–	27/09/14
M7	MF	21	4	–	–	14/08/14
M22	MF	–	–	15	4	21/01/16
M25	TR	–	–	4	2	13/11/15
M26	VW	–	–	6	1	29/10/15
F26	FU	–	–	3	2	27/05/15
Total		98	26	128	45	

Pack abbreviations are used (full names are listed in Additional file 1). The total number of traps in the last row is the number of different trap locations used during each session. The date of the last video capture is reported to evaluate persistence of focal animals supporting the assumption of population closure


Additional file 2:Reduced collection of videos showing the focal animals, filmed by camera traps and used for capture-recapture analysis. Available at https://figshare.com/s/f1b1146bc067683234d4. (MP4 813000 KB)


### Capture rate, spatial distribution and persistence of packs

The mean capture success over the whole period was 12.6 CE/100 trap-days, but that referring to the focal animals only was 6.4 and 5.0 CE/100 trap-days during the 2014 session and the 2015 session, respectively. All 14 focal animals were captured several times (3–21) during each session with the exception of F21 captured only once in 2014 (Table 2). Such high recapture rate, favoured by the marking behaviour, also resulted in captures at multiple traps: during the 2014 session 7 out of 10 wolves were captured at more than one trap (mean 2.9 traps/focal animal), while in the 2015 session, with a nearly double number of traps, all focal animals but one were filmed by several different cameras (mean 4.2 traps/focal animal). The spatial distribution of captures of each pack is shown in Fig. 1. The mean maximum distance moved by individuals captured at more than one trap was 8.16 ± 3.12 km (mean ± SD, *n* = 7) in 2014 and 7.56 ± 2.15 km (*n* = 11) in 2015. Population closure was supported by high persistence of focal animals during the study, confirmed by the subsequent monitoring in the area (see Table 2). Seven focal animals of the 2014 session and the three new ones observed in 2015 were still present at the end of the study period. The other four focal animals were observed almost until the end of each session or block (Table 2). The mean pack size was 3.40 ± 2.01 wolves (*n* = 10) excluding pups in summer 2014 and 4.17 ± 2.44 wolves (*n* = 12) in late winter-spring 2015. The overall intra-pack sex ratio was 1.12 (*n* = 53). Composition of each pack during the two sessions is reported in Additional file 1.

### Validation of video wolf recognition

During the study, 19 scats deposited by wolves in front of an active camera trap (“video-scats”) were collected and successfully genotyped. They produced 13 different genotypes corresponding to seven males and six females [34]. On the basis of video analysis, 10 wolves defecating at camera traps were classified as α individuals (see Additional file 1). Eight of them represented the α-pairs of three different packs: pair F1-M1 of PS pack, pairs F8-M7 (2014) and F9/10-M22 (2015) of MF pack, and pair F18-M15 of CN pack. Genetic analyses confirmed their identity and social rank, according to sampling/capture histories and parentage analysis, which confirmed their reproduction (Additional file 3). Similarly, another individual was recognized as the new α-male of the PN pack. Further video-scats confirmed the attribution of three individuals to their respective packs (see Additional file 1): a female (F5) and a dark-coated male (M3/My) were assigned by videos to PS pack and resulted to be the offspring of the PS pair; another defecating male (M27), was correctly identified as the new α-male of the PS pack, but it was not recognized as a previously video-captured β-male of the MF pack. Finally, an additional individual in a marginal territory (VC) was not recognized, and genetic analyses revealed that it was a previously unsampled daughter of the F7-M6 pair, dispersing from PN pack. To summarize, out of 19 successfully genotyped video-scats, in 15 events (79%) we were able to correctly recognize both the defecating individual and its pack. In three cases, we were able to recognize the pack but not the individual, and in just one case neither the pack nor the individual. Notably, no misidentification had consequences on intra-session capture histories, as status and pack of all focal animals were correctly assessed.

Individual recognition from videos was also validated by the achieved agreement among four different observers (Table 3). The expert observers 2 and 3 agreed with observer 1 in 85.0% of cases; the resulting capture histories were of 30 and 29 CEs respectively and were referred to 9 different packs, one pack less (MF pack in both cases) with respect to observer 1. The inexperienced observer 4 agreed with observer 1 in 80.0% of cases and produced a capture history of 28 CEs with 8 different packs (MF and LG packs were not identified). The highest agreement was obtained between observers 2 and 3, with 97.5% of CEs concordantly assigned cases, while the agreement between them and observer 4 was intermediate (90.0–92.5%). Inter-observer disagreement resulted in 25.0–35.2% underestimation of wolf pack density, but when discordant captures were excluded from the analysis, the difference dropped to 4.5–16.0% (Table 3).

**Table 3:** Results of inter-observer agreement test on wolf recognition. A selection of 40 videos was used for the tests. The number of capture events attributed by operators to a different wolf pack are reported

	Operator 1	Operator 2	Operator 3	Operator 4
PS -Poti South pack	5	9	8	8
MF - Monte Favalto pack	5	0	0	0
PNE –Poti North East pack	4	4	4	4
PN – Poti North pack	4	4	4	4
LG - Monte Lignano pack	2	2	2	0
VC –Val di Chio pack	2	2	2	3
CS –Catenaia South pack	2	2	2	2
CN –Catenaia North pack	2	2	2	2
VE –Vallesanta East pack	4	4	4	4
AT - Alto Tevere pack	1	1	1	1
Indeterminate	9	10	11	12
Total CE	40	40	40	40
Different Packs identified	10	9	9	8
Capture histories (nr. of CE assigned to a pack)	31	30	29	28
Matches with operator 1 (%)		34 (85.0)	34 (85.0)	32 (80.0)
Matches with operator 2 (%)			39 (97.5)	36 (90.0)
Matches with operator 3 (%)				37 (92.5)
Pack density (SD) estimated from full capture histories(model SPACECAP HN_TP)	1.56 (0.45)	1.14 (0.33)	1.17 (0.35)	1.01 (0.32)
Pack density (SD) estimated from only concordant capture histories(model SPACECAP HN_TP)		1.48 (0.47)	1.49 (0.46)	1.31 (0.44)

Operator 1 was experienced researcher contributing to study design and camera-trapping. Operators 2 and 3 were experienced field assistants in camera trapping, while operator 4 was inexperienced. Discrepancies among operators were solved by NGS data and confirmed the interpretation of operator 1. Hence, capture histories provided by operator 1 were used for model analysis. CE, capture event

### Wolf density

**Fig. 2:**
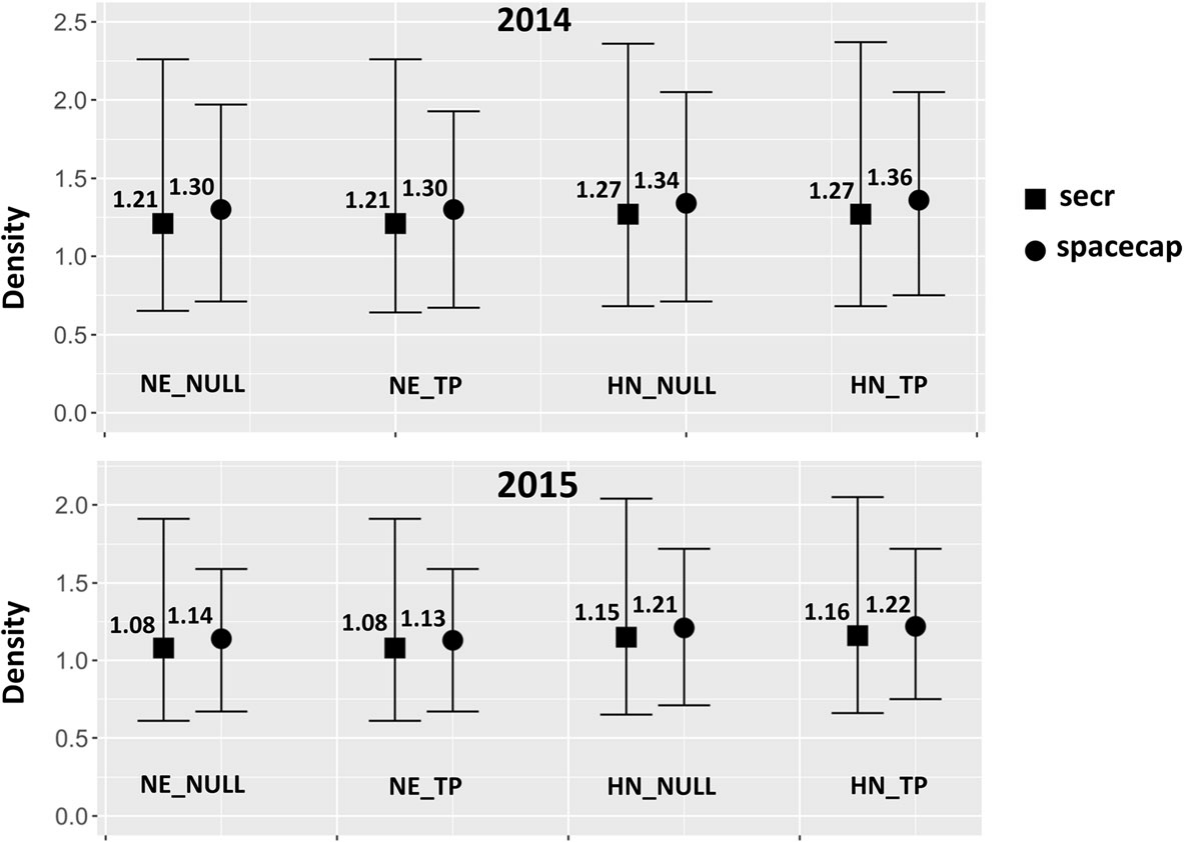
Comparison of wolf pack density estimates for the study area (Arezzo province, Italy) during 2014 and 2015 camera trap capture-recapture sessions, obtained by four models (NE-_NULL, NE_TP, HN_NULL and HN_TP, see text and Table 4 for details) using SPACECAP and *secr* estimators. Bars represent the 95% confidence intervals

Wolf pack density resulting from the Bayesian analysis in SPACECAP for the four tested models ranged between 1.30 ± 0.34 (SD) and 1.36 ± 0.35 packs/100 km^2^ in 2014, and between 1.13 ± 0.25 and 1.22 ± 0.26 in 2015 (Fig. 2). We chose not to include the covariate TRAP in our final Bayesian models on the basis of: *i*) the overlap between the confidence intervals of detection probability after (p2) and before (p1) the first capture (in the models including behavioural trap response); *ii*) the negligible difference in wolf pack density (0.1–1.0%) and goodness of fit between models with presence or absence of a behavioural trap response (session and detection function being equal); *iii*) an observed slight “trap happiness” effect, which was not expected and would be difficult to explain biologically for wolf in absence of baited traps (see [35, 36]). According to the Bayes *p*-value the best fitting models were negative exponential without trap response (NE_NULL) for session 2014 and half normal without trap response (HN_NULL) for 2015. The Bayes p-value close to 0.70 for 2014 suggested a good - but not optimal - model adequacy. Geweke’s diagnostic statistics showed convergence for all models. The analysis in *Secr* provided similar results: 1.21 ± 0.39–1.27 ± 0.41 packs/100 Km^2^ for the 2014 session and 1.08 ± 0.32–1.16 ± 0.34 for the 2015 session (Fig. 2). The best models with *secr* were the NE_NULL for session 2014 and HN_NULL for session 2015. A summary of the four selected models is given in Table 4.

**Table 4:** Summary of the best models explaining wolf pack density from spatially explicit capture-recapture data collected during sessions 2014 and 2015 (Arezzo province, Italy). a) parameters estimated by the Bayesian approach in SPACECAP; b) parameters estimated by the maximum likelihood approach in *secr*

a)								
Session	Model definition	Parameter	Posterior_Mean	Posterior_SD	95%_Lower_HPD_Level	95%_Upper_HPD_Level	z-score	Bayes p-value
2014	NE_NULL							0.714
		*σ*	2871.93	170.52	2565.85	3226.57	0.0404	
		λ_ο_	0.9028	0.4192	0.3154	1.7200	0.2870	
		Ψ	0.2880	0.0844	0.1358	0.4552	0.2071	
		Nsuper	31.20	8.14	17	47	−0.0156	
		Density	1.31	0.34	0.71	1.97		
2015	HN_NULL							0.609
		*σ*	2424.42	137.63	2181.91	2715.64	−0.8695	
		λ_ο_	0.1220	0.0176	0.0882	0.1573	0.9000	
		Ψ	0.2238	0.0590	0.1142	0.3393	0.2409	
		Nsuper	28.94	6.37	17	41	0.6992	
		Density	1.21	0.27	0.71	1.72		
b)								
Session	Model definition	Parameter	Mean	SE	95%_Lower_HPD_Level		95%_Upper_HPD_Level	
2014	NE_NULL	*σ*	1173.51	152.14	911.16		1511.4	
		g_o_	0.6879	0.2985	0.13		0.97	
		Density	1.21	0.4	0.64		2.26	
2015	HN_NULL	*σ*	2428.29	133.35	2180.665		2704.03	
		g_o_	0.1162	0.0156	0.0898		0.15054	
		Density	1.15	0.34	0.65		2.04	

NE and HN indicate, respectively, the negative exponential and half normal detection function. TP and NULL indicate, respectively, model with or without a behavioural trap effect as covariate. Density is expressed as number of wolf packs/100 km^2^. In SPACECAP the parameter σ is a “range parameter” of the species, λ_ο_ is the expected encounter frequency of an individual (i.e., focal animal) whose activity centre is exactly at trap location, Nsuper is the estimated number of individuals (i.e., focal animals) located in the state-space S, Ψ is the ratio between Nsuper and the maximum allowable number of individuals (i.e., focal animals) in S set by the user during data augmentation. Density is obtained dividing Nsuper by the surface of the state-space S. In *secr*, parameters *σ* and g_o_ are analogous to *σ* and λ_ο_ in SPACECAP

The increased trap density in 2015 seemed to have little to negligible effects on the parameter estimates and their variation. In fact, all parameters obtained for 2015 (48 traps, 560 km^2^ trap array) varied only slightly when they were calculated for the subset of 26 traps (414 km^2^ trap array) used in 2014. Pack density for the HN_NULL model was 1.22 ± 0.31 for the 26 traps array (λ_ο_ = 0.13 ± 0.02, *σ* = 2484 ± 186 m) and 1.21 ± 0.27 for the 48 traps array (λ_ο_ = 0.12 ± 0.02, σ = 2424 ± 138 m).

**Fig. 3:**
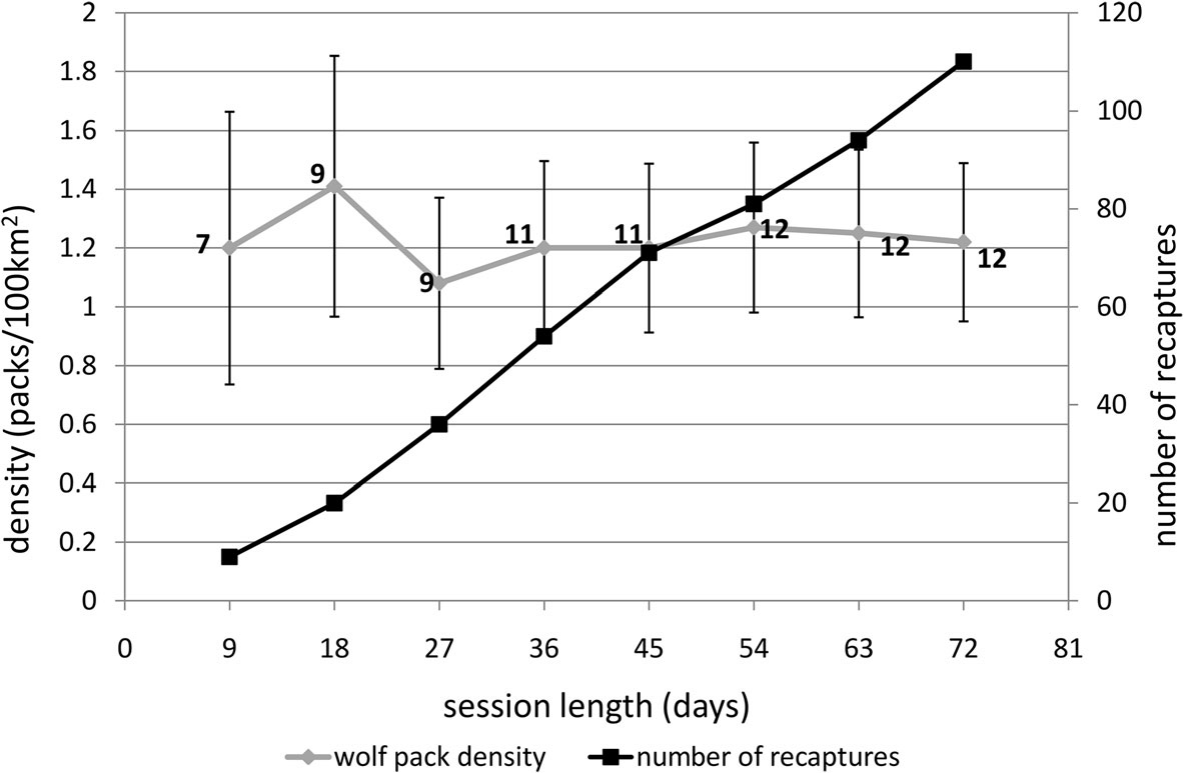
Effect of session length on wolf pack density estimates (grey line) and capture success (i.e., number of recaptures of focal animals, black line) in the study area (Arezzo province, Italy). Bars represent standard deviation and numbers near density estimates are the number of different packs detected

Session length analysis using HN_NULL model showed a stabilization of pack density estimate after 27 days, corresponding to a linear increase of number of recaptures with session duration followed by a plateau (Fig. 3).

Multiplying the pack density value obtained with the HN_NULL model in SPACECAP by the pack size estimated in each session as a conversion factor, we inferred a minimum wolf density of 4.40 wolves/100 km^2^ for summer 2014 (excluding pups and lone-transient wolves) and 5.04 wolves/100 km^2^ for late winter-spring 2015 (excluding lone-transient wolves).

## Discussion

### Wolf density estimate

Estimates of population size are crucial in conservation biology and population density represents a very useful indicator to compare situations across time and space. As regards the wolf, reliable density estimates require robust data about population size and the definition of the area where the individuals are being surveyed. Such data are usually obtained from radio-telemetry [37]. In order to achieve good estimates, particular attention should be paid to sampling effort and geographic scale, as these may affect values of wolf density and robustness of results. Indeed, population area definition is a key problem to convert numbers of individuals or packs into density, whatever is the methodology used for counting wolves. This issue may account for the scarcity of density data in the scientific literature, especially as regards areas where no animals were equipped with telemetry devices. Recently developed SCR models offer an alternative when live trapping of a sufficient amount of animals is infeasible. To our best knowledge, this is the first investigation applying SCR models to camera-trap data in wolves. Very recently, a similar SCR approach – but with Poisson models – was applied to NGS data to estimate wolf density and population size in a region of Spain [8]. The two studies offer two valuable alternatives to estimate wolf number in association to a non-arbitrary sampling area, thus providing robust density estimates. Our camera-based SCR approach provided a wolf density estimate of 1.21 ± 0.27 packs/100 km^2^ (assuming the 2015 full trap HN NULL as the most robust result) corresponding to a total wolf density of 5.04 individuals/100 km^2^ in late winter-spring, excluding extra-pack wolves. This is the first robust density estimate for a wolf population in Italy and one of the few available for Europe. Moreover, these values are among the highest ever recorded for this species (North-east Minnesota, [38]; Isle Royale in lake Michigan, [39]; Northern Range of Yellowstone National Park, [40]). There is strong evidence that wolf density is constrained by food supply, i.e. wild ungulates biomass [41, 42], although, at high prey density, social factors and intraspecific strife may play an important role in wolf survival and density limitation [25, 43, 44]. Using data collected from 32 study sites across North America, Fuller et al. [45] described a linear relationship between wolf density and ungulate biomass. Transforming the ungulate density estimates reported by Bassi and colleagues [33] for our study area into late winter biomass, we obtained a mean value of 710 kg/km^2^, corresponding to an ungulate biomass index of approximately 15 deer-equivalent [46], a higher value than those reported for North America. Although Fuller’s formula is not directly applicable to South European ecosystems because of the difference in wolf size, in energy requirement and in prey size and vulnerability, there is no doubt that the high productive broad-leaved forest ecosystems of Northern Apennines [47] can support the high wolf density described in this paper. In Italy, similar wolf density and pack size estimates were reported by Apollonio et al. [28] for the close Foreste Casentinesi National Park (4.7 wolves/100 km^2^, 4.2 wolves/pack) and by Ciucci & Boitani [28] for the Abruzzo-Lazio-Molise National Park (4–5 wolves/100 km^2^, 4.2 wolves/pack). Lower density estimates are generally reported for other European study areas, like the Costa da Morte in Spain (2.25 wolves/100 km^2^, [8]), the Bielowieza Primeval Forest (2.6 wolves/100 km^2^, 4.6 wolves/pack, [25]) and the Bieszczady National Park in Poland (3.3 wolves/100 km^2^, 3.9 wolves/pack, [48]), as well as in eastern Finland (0.2–0.4 wolves/100 km^2^, [26]), in agreement with the previously observed negative correlation between wolf density and latitude [25, 28].

### Prerequisites and adjustments to estimate wolf density by CT-based SCR models

Our results prove that a camera based SCR approach is suitable for wolf density estimates provided that some prerequisites are satisfied and methodological adjustments are implemented to successfully complete the study.

The main prerequisite is the presence of a sufficient amount of phenotypic variation in the population. In our study area, peculiar pelage colour patterns associated with introgressed individuals facilitated wolf recognition in five out of fourteen focal animals (Additional file 1). Other permanent natural marks, without genetic basis, accounted for four cases, while no evident peculiar phenotypic trait was present in the remaining five individuals. Nevertheless, in the latter case, individual recognition was possible by careful observation of both tail morphology (tail shape and length, hair length) and tail-raising pattern during scent-marking display (Additional file 1). The latter traits were of great importance, especially for the identification of wolves in nocturnal videos, where the assessment of coat colour pattern was difficult. An individual recognition relying on natural phenotypic cues was used in many studies on mammals that lack unambiguous marks, like chimpanzees, elephants, lions and red deer (for a review see [49]). These authors argued that natural idiosyncrasies present in each individual allow for individual recognition and may be even more reliable than artificial markings that are often lost during their life span. In agreement with this point of view, we believe that recognition of individuals in species lacking evident natural marks is possible if a sufficient amount of time is devoted to the identification of individual behavioural and physical cues.

The second issue concerns the validation of individual recognition, which is a crucial point for wolves, given the lack of unequivocal natural marks. Combining CT with NGS, we could verify an adequate subsample of our wolf identifications and disentangle partial inter-observer disagreement. In our test, pack assignments by the principal observer (LM) were confirmed by two expert operators in all cases but six (*n* = 34), and namely the captures of MF pack, which was not distinguished from the adjacent PS pack (but they were confirmed as two distinct packs by NGS). This 15% disagreement in individual recognition between expert operators produced a 25–27% underestimation of density estimates. However, this error strongly reduced (to 4–5%) when we discarded the discordant CEs from model analysis (Table 3), indicating that SCR analysis is more sensitive to geographic bias (e.g. merging two adjacent packs) than to a reduced capture history and/or to a smaller sample size. Accordingly, when validation is based on inter-observer agreement, we recommend including only concordant CEs in the capture history, as suggested by other authors (e.g., [50]).

When these prerequisites are satisfied, the classic CTCR method needs some adjustments to be successfully applied to wolf. First of all, the focus must be on packs instead of individuals. Three years of integrated camera trapping and NGS suggested that individual recognition is feasible for α individuals but not for all resident wolves. We were able to recognize α pairs of different packs by means of morphological and behavioural traits inasmuch as NGS validated all fifteen video assignments of ten α individuals belonging to four different packs (Additional file 1). Although we correctly assigned some β individuals to some given packs, we failed in tracking some between-packs movements of certain individuals and some changes in social status within packs that occurred during the study. However, our CR approach assumes no misidentification of α individuals only within a single session, i.e. few weeks or months, and not between years, so these limitations in individual recognition had no effect on capture history and density estimation. For a territorial group-living species like the wolf, referring to packs in population monitoring is more feasible and anyway well informative on total population size when adequate conversion factors - from packs to total individuals - can be applied [32].

Another challenging issue in density estimation using CR closed population models is to collect a sufficient sample size (i.e., number of packs and number of recaptures), since the precision of the estimation is low with small sample sizes [51]. When adopting one focal animal per pack as target for CR, the consequence is that the study area should be sufficiently large to encompass or intercept a minimum number of wolf packs. Taking into account Foster and Harmsen’s recommendations [51] and our results, we suggest ten packs as a possible threshold for an accurate estimation of pack density. As a consequence, the CTCR approach can be recommended in areas with high pack density, where it becomes more cost-effective than other methods like radio-tracking.

The last issue concerns study duration. In our approach, the actual CR session was a time window within a longer monitoring period, preceded by a pilot study necessary to collect preliminary video material for focal animal identification, to verify the presence of an adequate variability and to test for effectiveness of camera locations. Since focal animal identification is assisted by observation of wolves during scent-marking, checking putative camera station points as effective scent-marking sites is crucial to have a uniform detection probability. Moreover, an adjustment of camera position and settings is necessary to maximize captures and individual recognition. During our pilot study, we identified nine out of ten focal animals, that were captured by videos during the subsequent session 2014, and we also obtained a preliminary estimation of the animal movement parameter that led us to improve trap spacing in our sampling design. Similarly, our second CR session was followed by a control period to verify focal animal persistence and to collect additional data on pack size.

### Sampling design and model adequacy

Our sampling design proved to be very efficient. Indeed, we have a higher capture rate, and consequently number of recaptures, compared with most CR-based SCR studies on large and meso-predators [11, 13, 15, 16, 21, 22, 35, 50, 52–55]. Considering that our capture history was referred only to a single focal animal/pack and that our wolf density was in the range observed for other predators, we can speculate that both a predictable behaviour of the species and our CT strategy concurred to the high capture rate observed in our study. Notably, such high capture success could have provided similar results even with a smaller time investment. Indeed, based on our simulations, with the same settings, one month could be enough to obtain a sufficiently accurate pack density estimate (Fig. 3).

When comparing the results of *secr* and SPACECAP, we noticed higher estimates of density (up to 9%) and narrower confidence intervals for the latter (Fig. 2). The higher precision we obtained in SPACECAP would suggest relying on the Bayesian approach, which was also reported to be more robust in case of small number of individuals (see [35]).

Considering all the models we fitted, there was no clear indication in favour of a specific detection function. In fact, both the Bayesian (SPACECAP) and likelihood-based (*secr*) approaches pointed to a model with an exponential detection function for the 2014 session, while models with half normal detection function were selected by SPACECAP and *secr* for the 2015 session. The increased number of traps used in the latter and the consequent greater information on animal movements could possibly account for this discrepancy. In partial support of this interpretation, models with the exponential function were favoured for 2015, when the reduced trap array was used.

It is noteworthy that both SPACECAP and *secr* analyses were in a substantial agreement, pointing to models with the same detection function and without the trap covariate, thus making our findings even more robust.

On the basis of a larger number of recaptures and higher precision of the parameter estimates, overall, we considered the SPACECAP HN_NULL model for the 2015 session as the most reliable model, with an estimated density of 1.21/100 km^2^ and σ = 2424.4 m, corresponding to a 95% radius of about 5.9 km and a 95% use area of nearly 110 km^2^ (see [4]). These values are well in agreement with previous estimates of inter-pack distances [56, 57] and home range size [58, 59] in the Italian Apennines.

## Conclusions

Camera trapping and spatially explicit capture-recapture models, supported by NGS monitoring, offer a new approach for obtaining accurate wolf density estimates when capture and radio-tracking are not feasible. The application of this method to a species lacking evident natural marks like the wolf can be subdivided in the following operational steps:

i. a pilot CT study to verify the existence of sufficient phenotypic and behavioural individual variation, to train operators in video recognition, to check for used scent-marking sites, to estimate capture rate and sigma and to collect “video scats” for genetic validation;
ii. NGS monitoring to obtain genotypes of α individuals and other pack members to reconstruct parentage and pack structure;
iii. validation of focal animals recognition by matching CT and NGS results and by inter-observer agreement test of at least three different operators;
iv. actual CT session to record the capture history of a sufficient number of focal animals (ideally ten), with a suggested duration of 45 days.

Since this method needs an integrated use of CT and NGS, we believe it is applicable especially in existing monitoring programs, where these techniques are already applied [23, 60]. In such contexts, where a previous - although partial - knowledge of pack territory arrangement and genetic identity of some individuals exists, our method can provide outstanding data with a limited additional effort.

The application of this method can contribute to the achievement of lacking density data across Europe. The availability of robust and validated density estimates at local scale would allow an improvement of existing large-scale population estimates (e.g., [31, 61]) by providing reference density values particularly useful in countries without a national monitoring system, like Italy. Furthermore, the density of this top predator represents a key parameter in ecological studies on prey populations, and its availability over a large scale would enable interesting ecological comparisons across the wolf range.

## Methods

### Study area

The study was conducted in the Arezzo province, Central Italy, in an area of 560 km^2^ situated along a secondary ridge of the Apennine chain and delimited by the Arno and Tevere rivers to the east and west respectively, by the main Apennine ridge to the north and by the Chiana plain to the south (Fig. 1). Elevation ranges from 300 to 1414 m above sea level. Climate is temperate, with scarce snowfalls. Woods cover nearly 69% of the territory and are mainly composed of oaks (*Quercus pubescen*s and *Quercus cerris*), chestnut (*Castanea sativa*) and beech (*Fagus sylvatica*) with a varying composition depending on altitude. The wild ungulate community is dominated by wild boar (*Sus scrofa*) and roe deer (*Capreolus capreolus*) that are present with high density all over the area ([33]), while small numbers of fallow deer (*Dama dama*), red deer (*Cervus elaphus*) and mouflon (*Ovis orientalis musimon*) cluster in some areas only. Human settlements are scattered in the area, which is also adjacent to a large city (i.e., Arezzo, around 100,000 inhabitants, Fig. 1). Wolves live in mountainous and hilly parts of the area with frequent incursions in the plains, even close to human settlements. Resident wolf packs were found to have a low degree of relatedness with those of neighbouring areas: a finding that supports high pack stability and suggests a limited short-range dispersal across the two main rivers delimiting the study area [62].

### Camera trapping sampling design

A pilot camera trapping survey with an increasing number of camera traps (from 7 to 20) was performed from 1 April to 19 June 2014, with the following objectives: a) to mark (i.e. individually recognize) as many wolf packs as possible; b) to localise optimal camera trap sites; c) to perform a scenario analysis of trap spacing by a preliminary estimation of the animal movement parameter sigma (σ) and capture rate. More specifically, camera sites coincided with scent-marking sites used by wolves along dirty roads, mainly at crossroads, to maximize the detection probability and the permanence of wolves in front of the camera. We used three models of inbuilt HD digital cameras with passive infrared sensor (PIR) and LED flash, namely Bushnell trophy cam HD model 119477, UVision model UV 562 and UV 572. Cameras worked on “video mode” with a duration of 60 s. They were placed at 2 m of height to reduce possible vandalism, and were active 24/7. No bait was used to attract wolves. Each camera was visited at intervals from 2 to 20 days to change batteries and SD cards. This camera setting and location design was conceived to apply the CR method to a group-living species with limited natural marks, like the wolf, by observing scent-marking and social behaviour.

Subsequently, two sessions were carried out using information from the pilot study and comparing two different sampling designs. The first session in 2014 was a single-block of 26 single-camera locations. Trap distance (nearest neighbour) was 3.69 Km, corresponding to a 1.7 x σ optimal trap spacing [4, 63]. The trap array covered a minimum convex polygon of 414 km^2^. A sampling of 71 days (from 20 June to 28 August 2014) was chosen as a trade-off between the need to maximize the number of recaptures and that of assuming a demographically closed population [54]. A second session was carried out in 2015, increasing the number of camera points to 48, subdividing the study area into two blocks of 25 and 23 locations and enlarging the trap array to cover 560 km^2^. Trap distance was 2.74 km, with an optimal trap spacing of 1.1 x σ. Each block was surveyed for a total of 71 days between 10 January and 11 June 2015. Both sessions were planned to avoid the wild boar hunting season (September–December), during which trap vandalism and human-related mortality of wolves (i.e. poaching) are more frequent. After June 2015, camera trapping was opportunistically maintained with a reduced number of camera stations to collect data about persistence of individuals and pack size.

### Wolf classification

The wolf is a social species living in packs. The basic social unit is the α mating pair typically accompanied by their pups and, possibly, by other offspring born in the previous years [64]. We considered as a pack each social unit constituted by a territorial pair, regardless of the presence of any offspring in the group. α pairs were identified on the basis of their observed scent-marking behaviour, i.e. raised leg urination (RLU) and flexed leg urination (FLU), over-marking between male and female and ground scratching [65, 66]. For sake of simplicity, we classified as beta (β) all subordinate wolves older than 8 months associated to each α pair, and classified as pups the individuals born within the year and younger than 8 months (age they were supposed to reach by 31 December).

### Individual recognition of wolves/packs and pack size estimation by camera trap

Discrimination among α individuals of different packs was based mainly on the combination of unambiguous traits, like natural marks (i.e. blind eyes, pendant ears, fractures on tail or legs) and particular pelage colour patterns (dark coat, dark stripes, width of white mask, absence of black streaks on fore legs). Additionally, we used tail shape and posture during scent-marking display, that was often individual-specific (see Additional file 1). One recognizable individual of each α pair was selected to be the focal animal of the pack, and each time such focal individual was recognized in a video, such capture event was associated to the given pack. To ensure equal detectability among packs, a given α individual was identified in each capture event on the basis of its own traits only, disregarding the co-occurrence of other pack members and the pack size. The recognition of β individuals and pups, necessary to define size and composition of each pack, was allowed by individual-specific traits, if present, or by the association with a known α pair. Whenever possible, we assigned a video to a given pack on the basis of α, β and/or pups recognition. Whereas, when no resident wolf was recognized, or when recognized individuals were lone/transient wolves or non-territorial pairs, we classified the videos as indeterminate and excluded them from subsequent analyses. Finally, during each session, we estimated the size of each pack as the maximum number of wolves recorded in a single capture event.

### Inter-observer agreement

According to Foster and Harmsen [51], when monitoring species without evident natural markings, one has to clearly indicate the level of inter-observer agreement in individual recognition. Video analysis of all capture events and compilation of capture histories for model analysis were performed by a single operator (LM). However, to test the repeatability of our protocol, we selected two subsamples of 20 and 40 videos among the capture events collected during the 2014 survey, representative of most trapping sites. The first sample of 20 videos simulated the pilot survey and was intended as a training for inexperienced operators. The second group of 40 videos was used as the actual test. We tested the level of agreement between LM and three other operators, two of which were field assistants (EB, EF) and the third one (AC) was inexperienced both in field work and video analysis. The test was blind, operators having information only about location and date of video captures. Inter-observer agreement/disagreement was expressed as the percentage of matches in pack assignment and as the difference in the number of different packs identified by the three observers. We also evaluated the effect of inter-observer disagreement on density estimation by performing a model analysis of the 40 videos capture histories compiled by the different operators and by comparing differences in model outputs.

### Validation of individual wolf recognition by NGS

Our approach of density estimation by visual capture-recapture implies the recognition of single “focal animals” for each pack. Visual identification of wolves was validated by comparing individual recognition obtained by videos with non-invasive genetic identification of a subsample of wolves in the study area. In order to combine the two sources of information, a sample of scats (“video-scats”) deposited by wolves in front of an active camera trap was collected and genotyped. Defecating individuals were visually identified from videos by the first author (LM), while fecal samples were genotyped by NGB Genetics S.r.l. (Bologna, Italy). The laboratory genetic procedure is reported by Canu et al. [34] and can be summarized as follows: *i*) DNA was isolated from fecal material using a commercial kit; *ii*) 11 polymorphic autosomal microsatellites were PCR-amplified in triplicates following a multi-tube approach; *iii*) alleles were scored by running PCR products in an automatic sequencer; *iv*) consensus genotypes were reconstructed from replicated multilocus genotypes; *v*) sex was diagnosed genetically; *vi*) genotypes were compared with a database including all genotypes obtained during the ongoing long-term NGS monitoring in the area, with the aim to identify wolves already sampled in previous occasions; vii) all wolf genotypes obtained in the area were tested for parentage in order to identify mating pairs and their offspring for each sampling year.

Validation of individual recognition was achieved by comparing individual (and pack) assignment from videos with that deriving from NGS capture histories and parentage analysis. Accordingly, the α status was verified by the identification of compatible offspring in the sampling area. Finally, we compared spatial and temporal agreement between videos and genetic captures of the sampled focal animals.

### Spatially explicit capture-recapture models

To obtain an estimate of wolf density in our study area, we adopted a class of capture-recapture models that incorporate the information about the spatial location of both individuals and camera traps. These spatially-explicit capture-recapture (SCR) models have the advantage of providing density estimates that do not depend on a heuristic definition of the sampled area [4]. The SCR approach implies some important assumptions: a) all individuals are recognizable; b) individuals are encountered independently from one another; c) encounters of the same individual are independent. Since we were not able to identify all wolves of our population and most captures concerned social groups (where captures of different wolves are correlated), we constructed capture histories of packs instead of individuals, selecting one focal animal for each pack, almost always an α individual. We then inferred the wolf population density by using the estimated pack density and an estimate of the mean pack size as conversion factor [32].

To estimate wolf pack density we adopted two different SCR approaches: the Bayesian SCR models implemented in the R package SPACECAP 1.1. [67] and the maximum likelihood-based estimator implemented in the R package *secr* 2.10.4 [68]. Both packages require 3 different input files: a state-space file, a trap deployment file and an animal capture file. The state-space file (i.e., all possible locations of the wolf pack activity centres) consisted in a grid of 6433 regularly spaced (666 m) points buffering the trap array by a 15-km radius to include the home ranges of all the captured packs. Each point of the state-space was categorized as “habitat” when suitable for the species (83.5%), or “non habitat” when unsuitable (i.e., water bodies, towns, villages and open cultivated plain, 16.5%). Only “habitat” points were finally retained in the state-space, covering a total surface of 2382 km^2^. Then we compiled the trap deployment files, indicating, for each session, the activity time of each camera trap during the k sampling occasions, and the capture history files, indicating which focal wolf was captured at which camera trap on a given sampling occasion). The duration of each sampling occasion was 24 h, starting from 3 p.m. to 2.59 p.m. of the subsequent day, to solve for the “midnight problem” [69]. We defined as independent capture event (hereafter CE) a single capture/trap/day of a focal individual (independently of the number of videos recorded). The SCR models were specified with a Bernoulli encounter process, in which a pack could only be detected once in each trap per sampling occasion.

In both SPACECAP and *secr*, for each session (2014 and 2015), we tested two different encounter probability models (the HN and the NE detection functions) and the effect of trap-specific response as a covariate, to accommodate possible changes in individual encounter probability after the first detection (e.g., in case of behavioural effects, the so-called “trap happiness” or “trap shyness”). We tested the presence of trap covariate, expecting either no effect or a little negative effect (trap-shyness), since no bait was used on the site and no flash was used for nocturnal videos. Therefore, four models were fitted for each session: two of them with trap as a covariate (HN_TP and NE_TP) and the remaining two without covariates (HN_NULL and NE_NULL), for a total of 8 models Additional file 4. The SPACECAP analyses were performed with a total of 100,000 iterations, a burn-in of 5000 iterations and a thinning rate of 10. The z-score statistic based on Geweke’s diagnostic was used to evaluate convergence. When values of [z-score] were > 1.6 for one or more parameters then new MCMC chains were run until convergence was reached. The program uses uniform prior distributions for all parameters (from 0 to ∞ for positive parameters, from -∞ to ∞ otherwise). For further details on the implemented algorithm and default settings see [67].

Model selection in the Bayesian framework is not straightforward, and is often carried out using the Deviance Information Criterion (DIC), but recent literature warns against its effectiveness, since this analysis requires a very careful tuning not to be misleading [4, 70]. Given the presence of a single fixed effect (i.e., trap effect) in our set of models, in agreement to [4], we adopted a pragmatic approach to evaluate the inclusion of this covariate in our final model. Specifically, we considered, for a given detection function and session: i) the effect of the inclusion of the covariate on the estimated parameters of interest (e.g., density) and on the goodness of fit of the model; ii) the overlap between the confidence interval of the estimated encounter probability after the first capture (p2) and before the first capture (p1). As regards the choice of the best detection function and the evaluation of model adequacy, the Bayesian *P*-value based on individual encounter frequencies was used [4], choosing as the best fitting model that with Bayes *p*-value closest to 0.5. For the likelihood-based analysis in *secr* the best model for each session was selected by considering the Akaike’s information criterion with the correction for small sample sizes (lower AICc).

Furthermore, using the best model selected in SPACECAP for session 2015 as base model, we tested for trap density effect, i.e., whether the increase in trap number in 2015 significantly influenced the parameter estimates and their variation. To the purpose, we analysed the data of the 2015 session with a reduced number of traps equalling those used in 2014 (i.e., 26) and then compared the results with that obtained with the whole trap set. Similarly, we tested for the effect of session length, and consequently of the number of recaptures, in model parameters estimate by creating eight capture histories of increased duration (9, 18, 27, 36, 45, 54, 63 and 72 days), adopting, also in this case, the best combination of covariates/detection function.

## Additional files

Additional file 1: Individuals identified in the study area during the two sessions of camera trapping in 2014 and 2015. (XLSX 66 kb)

Additional file 3: Reconstructed pedigrees of some monitored packs in the study area, detected by camera trapping and non-invasive genetic sampling. (PDF 410 kb)

Additional file 4: Performance of SPACECAP and *secr* models and model selection. (PDF 378 kb)

Additional file 5: Datasets and R scripts used for SPACECAP and *secr* models. Datasets include capture histories, trap histories and state-space. (RAR 45 kb)

## Abbreviations

CE: Independent capture event
CR: Capture-recapture
CT: Camera trapping
CTCR: Camera trap capture-recapture
HN: Half-normal (detection function)
NE: Negative exponential (detection function)
NGS: Non-invasive genetic sampling
SCR: Spatially explicit capture-recapture
SD: Standard deviation
