# Supplementary material for: Estimation of pack density in grey wolf (Canis lupus) by applying spatially explicit capture-recapture models to camera trap data supported by genetic monitoring
Source: Front Zool. 2018 Oct 3;15:38. doi: 10.1186/s12983-018-0281-x (PMC6171198; doi:10.1186/s12983-018-0281-x)
Supplement: Supplementary file 3 — Reconstructed pedigrees of some monitored packs in the study area, detected by camera trapping and non-invasive genetic sampling. (PDF 410 kb) [file 12983_2018_281_MOESM3_ESM.pdf]

Estimation of pack density in grey wolf (*Canis lupus*) by applying spatially explicit capture-recapture models to camera trap data supported by genetic monitoring

by Luca Mattioli, Antonio Canu, Daniela Passilongo, Massimo Scandura, Marco Apollonio

Additional file 3 - History and composition of the four packs with at least one individual genotyped (video-scats), as resulting from combination of CT and NGS data. The period 2014-2016 is considered in order to include the two CT sessions and genetic data confirming the reproduction or replacement of alpha individuals. Males are represented by squares, females by circles, and individuals of unknown sex by hexagons (J = pup). Alpha individuals have a black border and are connected to their mate by black lines. Full coloured polygons correspond to wolves whose identity was confirmed by videoscats (the lower alphanumeric code indicates the genotype). Alpha individuals that were genotyped by NGS but not videoscats are indicated by an asterisk. Vertical dashed lines indicate the observation period covered by CT. Years refer to the period comprised between 1st May (year X) and 30th April (year X+1).

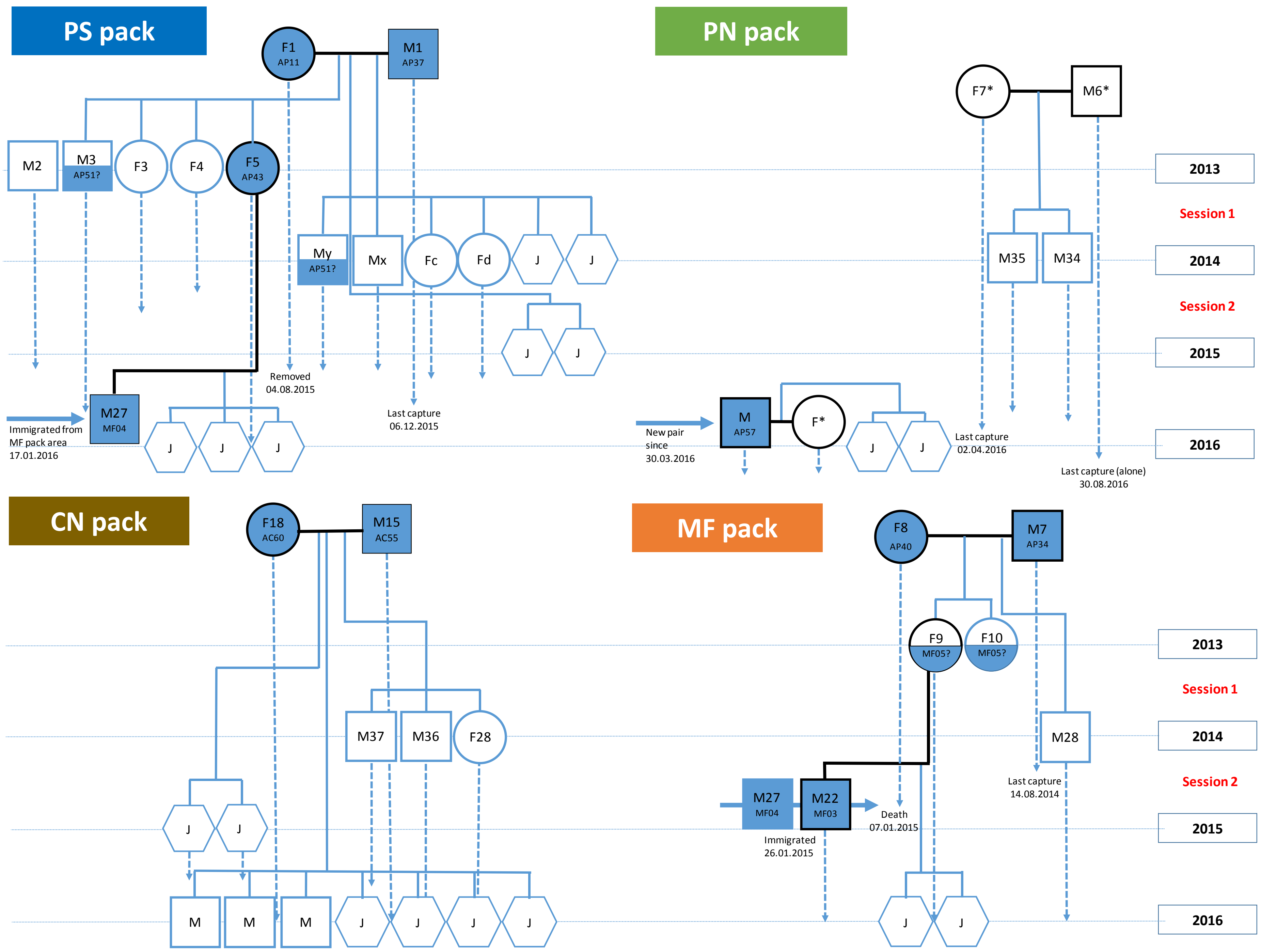

PS pack
PN pack
CN pack
MF pack
F1 AP11
M1 AP37
M2
M3 AP51?
F3
F4
F5 AP43
My AP51?
Mx
Fc
Fd
J
Removed 04.08.2015
Last capture 06.12.2015
Immigrated from MF pack area 17.01.2016
M27 MF04
F7*
M6*
M35
M34
New pair since 30.03.2016
M AP57
F*
Last capture 02.04.2016
Last capture (alone) 30.08.2016
2013
Session 1
2014
Session 2
2015
2016
F18 AC60
M15 AC55
M37
M36
F28
M
F8 AP40
M7 AP34
F9 MF05?
F10 MF05?
M28
Last capture 14.08.2014
M27 MF04
M22 MF03
Death 07.01.2015
Immigrated 26.01.2015
