# Supplementary material for: Estimation of pack density in grey wolf (Canis lupus) by applying spatially explicit capture-recapture models to camera trap data supported by genetic monitoring
Source: Front Zool. 2018 Oct 3;15:38. doi: 10.1186/s12983-018-0281-x (PMC6171198; doi:10.1186/s12983-018-0281-x)
Supplement: Supplementary file 4 — Performance of SPACECAP and secr models and model selection. (PDF 378 kb) [file 12983_2018_281_MOESM4_ESM.pdf]

Estimation of pack density in grey wolf (*Canis lupus*) by applying spatially explicit capture-recapture models to camera trap data supported by genetic monitoring

by Luca Mattioli, Antonio Canu, Daniela Passilongo, Massimo Scandura, Marco Apollonio

Additional file 4 - Performance of SPACECAP and *secr* models and model selection

a) SPACECAP

Model	Year	Covariates	Detection function	Nr. of parameters	Bayesian p-value
NE_TP	2014	trap-specific effect	exponential	4	0,703
NE_NULL	2014		exponential	3	0,714
HN_TP	2014	trap-specific effect	halfnormal	4	0,766
HN_NULL	2014		halfnormal	3	0,805
HN_TP	2015	trap-specific effect	halfnormal	4	0,591
HN_NULL	2015		halfnormal	3	0,609
NE_TP	2015	trap-specific effect	exponential	4	0,642
NE_NULL	2015		exponential	3	0,676

b) *secr*

Model	Year	Equation	Covariates	Detection function	Nr. of parameters	Log likelihood	AICc	ΔAICc	W*i*	Cumul. W*i*	Evidence ratio
NE_NULL	2014	D~1 g0~1 sigma~1		exponential	3	-386,094	782,187	0,000	0,8735	0,873	
HN_NULL	2014	D~1 g0~1 sigma~1		halfnormal	3	-388,555	787,110	4,923	0,0745	0,948	11,721
NE_TP	2014	D~1 g0~bk sigma~1	trap-specific effect	exponential	4	-386,014	788,027	5,840	0,0471	0,995	18,542
HN_TP	2014	D~1 g0~bk sigma~1	trap-specific effect	halfnormal	4	-388,281	792,561	10,374	0,0049	1,000	178,958
HN_NULL	2015	D~1 g0~1 sigma~1		halfnormal	3	-559,335	1127,670	0,000	0,6923	0,692	
HN_TP	2015	D~1 g0~bk sigma~1	trap-specific effect	halfnormal	4	-557,793	1129,299	1,629	0,3066	0,999	2,258
NE_NULL	2015	D~1 g0~1 sigma~1		exponential	3	-565,984	1140,968	13,298	0,0009	1,000	772,012
NE_TP	2015	D~1 g0~bk sigma~1	trap-specific effect	exponential	4	-564,968	1143,650	15,980	0,0002	1,000	2951,297

D=density; D~1 indicates constant density; g0 is the baseline encounter probability; g0~1 indicates the intercept-only (i.e., constant) model; g0~bk indicates the local trap response model; sigma: spatial scala parameter (determining how rapidly capture probability declines with distance)
